# Supplementary material for: Changes in Smoking Behaviour and Home-Smoking Rules during the Initial COVID-19 Lockdown Period in Israel
Source: Int J Environ Res Public Health. 2021 Feb 17;18(4):1931. doi: 10.3390/ijerph18041931 (PMC7922914; doi:10.3390/ijerph18041931)
Supplement: Supplementary file 1 [file ijerph-18-01931-s001.pdf]

<u>Supplemental file 1:</u> Full survey questionnaire (translated from Hebrew to English)

For the exact wording used in Hebrew please email the corresponding author

Greetings,

We are conducting a survey exploring the changes in smoking habits and other health habits among current and former smokers during the COVID-19 pandemic period. This is an anonymous survey filled out through an electronic questionnaire. We, the researchers, do not have access to any personal data about the participants. You are free to decide not to answer any of the questions in the questionnaire and to stop answering the questionnaire at any time. The duration of the questionnaire is approximately 20 minutes.

Answering the questionnaire constitutes informed consent on your part to participate in the survey. For any questions or problems related to filling out this questionnaire, please contact the Chief Investigator, Dr. Yael Bar-Zeev, at Yael.Bar-Zeev@mail.huji.ac.il

Thank you for your cooperation,

Dr. Yael Bar Zeev

Public Health Physician and Senior Lecturer

School of Public Health

Hebrew University – Hadassah

Do you agree to participate in this survey? Yes / No (end survey)

1. Do you currently smoke cigarettes?
   a) Yes, every day (move to question 3)
   b) Yes, but only occasionally (move to question 3)
   c) No, but I smoked in the past and quit (move to question 2)
   d) No, I have never smoked (end survey)

2. (If they answered “c” for question 1): When did you quit smoking?
(afterwards, skip to question 17)
   a) Since the COVID-19 pandemic period began
   b) 3-12 months ago (before the COVID-19 pandemic period began, but less than a year ago)
   c) I have never smoked (end survey)

3. (If they answered “a” or “b” for question 1): **Before** the COVID-19 pandemic period in Israel, on average, how many cigarettes did you smoke a day? ___________

4. Before the beginning of the COVID-19 pandemic period in the Israel, how long did it take you from the time you woke up in the morning until you smoked your first cigarette?
   a) Within 5 minutes
   b) 6-30 minutes
   c) 31-60 minutes
   d) Over an hour

5. In your opinion, since the COVID-19 pandemic period began in Israel:
   a) I smoke more than usual
   b) I smoke the same amount as usual
   c) I smoke less than usual

6. Since the COVID-19 pandemic period began in Israel, how many cigarettes do you smoke on average per day? ___________

7. **Before** the beginning of the COVID-19 pandemic period in Israel, how do you estimate your level of motivation to quit smoking was, from 1-10 (1-did not want to quit smoking at all; 10-very much wanted to quit smoking)

1 2 3 4 5 6 7 8 9 10

8. In your opinion, has your motivation to quit smoking changed since the COVID-19 pandemic began in Israel?
   a) Yes, it rose considerably
   b) Yes, it rose slightly
   c) No, it remained exactly the same
   d) Yes, it dropped slightly
   e) Yes, it dropped significantly

9. **Before** the beginning of the COVID-19 pandemic period in Israel, how much do you estimate that you felt you were able to quit smoking from 1-10 (1-did not feel able to quit at all; 10 felt very able to quit)

1 2 3 4 5 6 7 8 9 10

10. In your opinion, has the feeling of being able to quit smoking changed since the COVID-19 pandemic period began in Israel?

a) Yes, it rose considerably
b) Yes, it rose slightly
c) No, it remained exactly the same
d) Yes, it dropped slightly
e) Yes, it dropped significantly

11. Do you feel that since the beginning of the COVID-19 pandemic period in Israel, the frequency of urges to smoke has increased (i.e. the number of times you feel during the day that you want to smoke)?

a) Yes, it rose considerably
b) Yes, it rose slightly
c) No, it remained exactly the same
d) Yes, it dropped slightly
e) Yes, it dropped significantly

12. Do you feel that since the beginning of the COVID-19 pandemic period in Israel, the strength of the urges to smoke has increased?

a) Yes, it rose considerably
b) Yes, it rose slightly
c) No, it remained exactly the same
d) Yes, it dropped slightly
e) Yes, it dropped significantly

13. Have you made any attempts to quit smoking since the COVID-19 pandemic period began in Israel?
Yes / No

14. How many of these attempts have lasted more than 24 hours? ____________

15. What is the longest amount of time you have been able to quit smoking and remain abstinent since the COVID-19 pandemic period began?

a) Less than 24 hours
b) 1-3 days
c) 4-7 days
d) 8-14 days
e) Over two weeks

16. During the COVID-19 pandemic period, did you use any of the following measures to support a smoking cessation attempt? (All correct answers can be marked)

a) Telephone consultation through HMO / national Ministry of Health quit-line Yes / No
b) Family doctor support Yes / No
c) Prescription medication treatment Yes / No
d) Over-the-counter medication treatment Yes/ No
e) Other: _______________ Yes / No
f) I did not use any means of smoking cessation support Yes / No

17. **__Before__** the COVID-19 pandemic period, what were your home rules regarding smoking?

a) Smoking is allowed throughout the house
b) Smoking is only allowed in some rooms
c) Smoking is only allowed in one particular room
d) Smoking is only allowed on the balcony
e) Smoking is not allowed anywhere

18. Now, during the COVID-19 pandemic period, what are the rules in your home regarding smoking?

a) Smoking is allowed throughout the house
b) Smoking is only allowed in some rooms
c) Smoking is only allowed in one particular room
d) Smoking is only allowed on the balcony
e) Smoking is not allowed anywhere

19. Do you smoke and / or use any other tobacco and / or smoking products?

a) Electronic cigarette Yes / No
b) Heated tobacco products such as iQOS Yes / No
c) Hookah Yes / No
d) Other: ________ Yes / No

20. Do you suffer from any chronic diseases (including heart disease / chronic obstructive pulmonary disease such as bronchitis or emphysema / asthma / hypertension / diabetes / cancer)? Yes / No

21. In your opinion, is the risk of smokers getting infected with the COVID-19 virus different from the risk of non-smokers?

a) No, smokers and non-smokers have the same risk of contracting the COVID-19 virus
b) Yes, the risk of smokers being infected with the COVID-19 virus is **__higher__**
c) Yes, the risk of smokers being infected with the COVID-19 virus is **__lower__**

22. In your opinion, is the risk of smokers experiencing a more severe case of COVID-19 (if infected) different from the risk of people who do not smoke?

a) No, smokers and non-smokers have the same risk of experiencing a more severe case of COVID-19 (if infected)
b) Yes, smokers' risk of experiencing a more severe case of COVID-19 (if infected) is **__higher__** than non-smokers.

c) Yes, smokers' risk of experiencing a more severe case of COVID-19 (if infected) is **lower** than non-smokers.

23. How much do you estimate your risk of being infected with the corona virus from 1-10, with 1 being not likely to be infected at all and 10 being sure to be infected with the COVID-19 virus.

1 2 3 4 5 6 7 8 9 10

24. How much do you estimate your risk experiencing a more severe case of COVID-19 (if infected) from 1-10, with 1 being not likely to experience a more severe case and 10 being sure I will experience a more severe case.

1 2 3 4 5 6 7 8 9 10

25. In a normal week, **before** the COVID-19 pandemic period, how much cumulative time do you spend exercising?

a) I do not devote any time to physical activity
b) Up to 30 minutes a week
c) 30-90 minutes a week
d) 90-150 minutes
e) Over 150 minutes

26. Since the COVID-19 pandemic period in Israel began, has there been a change in the time you devote to physical activity?

a) Yes, it rose considerably
b) Yes, it rose slightly
c) Has not changed
d) Yes, it dropped slightly
e) Yes, it dropped significantly

27. What is true about your eating habits **before** the COVID-19 pandemic period? (You can answer more than one answer)

a) Trying to eat regular meals every day	Yes / No
b) Trying to reduce salty and / or sweet snacks	Yes / No
c) Trying to reduce sugary drinks	Yes / No
d) Trying to eat at least 5 or more portions of fruit and vegetables a day	Yes / No

28. Has there been any change in your eating habits since the COVID-19 pandemic period began in Israel?

a) Eating regular meals	has not changed / less good now / better now
b) Eating salty and / or sweet snacks	has not changed / less good now / better now
c) Sugary drinks	has not changed / less good now / better now
d) Eating fruits and vegetables	has not changed / less good now / better now

29. **Before** the COVID-19 pandemic period, to what extent did you feel mentally stressed?

a) To a very small extent
b) To a small extent
c) Moderate
d) To a large extent
e) To a very large extent

30. Since the COVID-19 pandemic period began, to what extent do you feel that your degree of mental stress has changed?
   a) Rose considerably
   b) Rose slightly
   c) Has not changed
   d) Decreased slightly
   e) Significantly decreased

31. **Before** the COVID-19 pandemic period, what was your employment status?
   a) Full-time employee
   b) Permanent part-time employee
   c) Temporary employee (for example by hours)
   d) Self-employed
   e) Not working (including unemployed)
   f) Retired

32. Has your employment status changed as a result of the COVID-19 pandemic?
   a) No
   b) Yes, I lost my job
   c) Yes, I was placed on unpaid leave
   d) Yes, I was fired
   e) Yes, my income as self-employed has been significantly cut

33. What is your gender? Male / Female

34. Year of birth: ______________

35. What is your level of education?
   a) Less than 12 years of schooling
   b) I completed 12 years of schooling
   c) Bachelor's degree
   d) Master's degree or higher

36. Religion:
   a) Jewish
   b) Muslim
   c) Christian
   d) Other _________

37. Marital status:
   a) Single
   b) Living with a partner
   c) Married
   d) Widower
   e) Divorced

38. How many adults (over the age of 18) live with you at home? _________

39. How many children (under the age of 18) live with you at home? ___________

40. What is the age of the youngest child living with you at home? ____________ years

41. Are there any other smokers living with you in the house? Yes / No

42. If so, how many other smokers live with you at home? ___________

43. Are any of the people living with you at home at high risk of experiencing a more severe case of COVID-19 (if infected) (old age or have an underlying chronic illness)? Yes / No

44. What type of residence do you currently reside in?
   a) House with a garden
   b) Apartment with a balcony
   c) Apartment without a balcony
   d) Other: ______________

45. What is your current situation regarding the COVID-19 virus?
   a) I was not infected with the COVID-19 virus and to the best of my knowledge was not exposed to someone with COVID-19
   b) I was not infected with the COVID-19 virus, but I was exposed to someone with COVID-19 and I am now in quarantine
   c) I was not infected with the COVID-19 virus, but I was exposed to someone with COVID-19, was in quarantine and am already out of quarantine
   d) I was infected with the COVID-19 virus and I am currently hospitalized
   e) I was infected with the COVID-19 virus and I am currently in isolation in a hotel or at home
   f) I was infected with the COVID-19 virus and was previously hospitalized or in isolation

Thank you for participating in this survey.
If you are interested, you can contact free smoking cessation support at the Ministry of Health's national quit line - *6800.
For any questions or problems related to completing this questionnaire, you can contact the Principal Investigator:
Dr. Yael Bar-Zeev
Email- Yael.Bar-Zeev@mail.huji.ac.il
